# Using the big five inventory to evaluate the personality traits of medical staff

**DOI:** 10.1192/j.eurpsy.2025.1931

**Published:** 2025-08-26

**Authors:** N. Rmadi, A. Hrairi, I. Ben Hnia, O. Walha, N. Kotti, M. Hajjaji, K. Jmal Hammami

**Affiliations:** 1 Occupational medicine department, Hedi chaker university hospital, University of Sfax; 2 familiy medicine department, University of Sfax, Sfax, Tunisia

## Abstract

**Introduction:**

Personality traits are enduring and stable characteristics that reflect an individual’s behaviour, thoughts and feelings. Research indicates that specific traits can affect not only the well-being of healthcare professionals but also their interactions with colleagues and patients.

**Objectives:**

This study aims to assess the personality traits of healthcare professionals using the Big Five Inventory (BFI).

**Methods:**

A descriptive cross-sectional study was conducted among healthcare personnel who consulted the department of occupational medicine of Hedi Chaker Hospital of Sfax to September 2024. The survey was conducted through a self-questionnaire that included sociodemographic data, lifestyles. We also used BFI which measures the Big Five personality traits through five key dimensions: extraversion, agreeableness, conscientiousness, neuroticism and openness.

**Results:**

The study involved 41 consultants (12 men and 29 women) with an average age of 31.2 ± 7.4 years. An urban origin was identified in 87.8% of the cases. Medical staff scored 3.36 ± 0.52 for extraversion, 3.21 ± 0.56 for agreeableness, 3.22 ± 0.61 for conscientiousness, 3.01 ± 0.78 for neuroticism and 3.2 ± 0.65 for openness. A significant association was found between neuroticism and urban versus rural origin (p=0.001). Moreover, associations were found between BFI dimensions: agreeableness with extraversion (p=0.007, r=0.41) and openness (p=0.002, r=0.46).

**Conclusions:**

This study highlights the importance of assessing personality traits among healthcare professionals. Understanding these personality dimensions can provide valuable insights for improving workplace dynamics, enhancing team collaboration, and ultimately fostering better patient care outcomes.

**Disclosure of Interest:**

None Declared

